# Monomorphic Ventricular Tachycardia as a Presentation of Giant Cell Myocarditis

**DOI:** 10.1155/2019/7276516

**Published:** 2019-06-19

**Authors:** Michael H. Chiu, Cvetan Trpkov, Saman Rezazedeh, Derek S. Chew

**Affiliations:** Libin Cardiovascular Institute of Alberta, Cummings School of Medicine, University of Calgary, Alberta, Canada

## Abstract

**Background:**

Idiopathic giant cell myocarditis (GCM) has a fulminant course and typically presents in middle-aged adults with acute heart failure or ventricular arrhythmia. It is a rare disorder which involves T lymphocyte-mediated myocardial inflammation. Diagnosis is challenging and requires a high index of suspicion since therapy may improve an otherwise uniformly fatal prognosis.

**Case Summary:**

A previously healthy 54-year-old female presented with hemodynamically significant ventricular arrhythmia (VA) and was found to have severe left ventricular dysfunction. Cardiac MRI demonstrated acute myocarditis, and endomyocardial biopsy showed giant cell myocarditis. She was treated with combined immunosuppressive therapy as well as guideline-directed medical therapy. A secondary prevention implantable cardioverter defibrillator (ICD) was implanted.

**Discussion:**

GCM is a rare, lethal myocarditis subtype but is potentially treatable. Combined immunosuppression may achieve partial clinical remission in two-thirds of patients. VA is common, and patients should undergo ICD implantation. More research is needed to better understand this complex disease.

**Learning Objectives:**

Giant cell myocarditis is an incompletely understood, rare cause of myocarditis. Patients present predominately with heart failure and dysrhythmia. Diagnosis is confirmed by histopathology, and immunosuppression may improve outcomes. ICD implantation should be considered. In the absence of treatment, prognosis is poor with a median survival of three months.

## 1. Introduction

Idiopathic giant cell myocarditis is a rare clinical entity first recognized in the early 1950s [1]. GCM has a fulminant course with an autoimmune pathophysiology in virus-negative GCM [2, 3]. Often fatal due to arrhythmia or heart failure, two-thirds of patients exhibit response to immunosuppressive therapy, and some undergo cardiac transplantation [2]. We report a case of a previously healthy female diagnosed with idiopathic giant cell myocarditis.

## 2. Case Report

A previously healthy 54-year-old female presented to emergency services with acute onset of malaise, nausea, palpitations, and presyncope. Her ECG showed monomorphic ventricular tachycardia at 230 bpm, and she underwent successful cardioversion. Hemodynamic stability was restored, and she was admitted to the cardiac intensive care unit.

Initial cardiovascular examination was pertinent for a positive abdominojugular reflux sign and a third heart sound. There was no clinical evidence of pulmonary or systemic congestion or low cardiac output state, and no other manifestations of systemic disorders were present.

**Figure 1:**
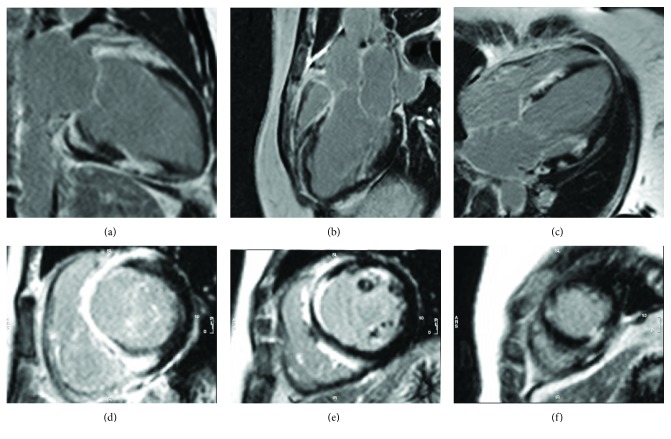
Cardiac myocardial resonance imaging demonstrating late gadolinium enhancement of the basal to midanterior, anteroseptal, inferoseptal, and inferior segments and apical inferior segments of the left ventricle. Patchy enhancement within the septum on the side of the ventricle: (a) 2-chamber view (b) 3-chamber view (c) 4-chamber view, (d) short-axis view at the base, (e) short-axis view at the level of the papillary muscles, and (f) short-axis view at the level of the apex.

ECG in sinus rhythm revealed a nonspecific intraventricular conduction delay with a QRS duration of 130 ms, a P wave of 1 mm in the lead II, a PR interval of 166 ms, and a QTc of 507 ms at a heart rate of 96 bpm. Transthoracic echocardiography showed left ventricular systolic dysfunction with an estimated ejection fraction of 35%, preserved right ventricular function, and no valvular abnormalities (aortic root dimension of 2.9 cm, left atrium of 3.2 cm, LV diastole of 4.9 cm, LV systole of 4.0 cm, fractional shortening of 18.6%, interventricular septum of 0.85 cm, posterior wall of 0.78 cm, left atrium volume index of 36.3 ml/m^2^, left ventricular mass of 79.3 grams/m^2^, left ventricular outflow tract diameter of 2.2 cm, stroke volume of 39.9 ml, end diastolic volume (MOD-bp) of 128.5 ml, ejection fraction (MOD-bp) of 31.1%, cardiac output (LVOT) of 4.9 l/min, stroke volume (LVOT) of 57.3 cc, TAPSE of 2.2 cm, and RV S' velocity of 11.5 cm/sec). Coronary arteries were angiographically normal. On cardiac magnetic resonance imaging, there was extensive, midwall patchy late gadolinium enhancement consistent with acute myocarditis (Figure 1).

A serum blood work revealed a hemoglobin count of 141 g/l with an MCV of 92 fl, a platelet count of 182 × 10^9^/l, a WBC of 12.3 × 10^9^/l with a differential (neutrophil 8.2 × 10^9^/l, lymphocytes 2.8 × 10^9^/l, monocytes 0.9 × 10^9^/l, eosinophils 0.3 × 10^9^/l, and basophils 0.1 × 10^9^/l), a high-sensitivity troponin T of 46 ng/l, an NT-proBNP of 261 ng/l, an ESR of 11 mm/h, and a CRP of 3.2 mg/l. An infectious panel was negative for cytomegalovirus, Epstein–Barr virus, hepatitis B, hepatitis C, herpes simplex virus, HIV, mumps, toxoplasmosis, and varicella.

**Figure 2:**
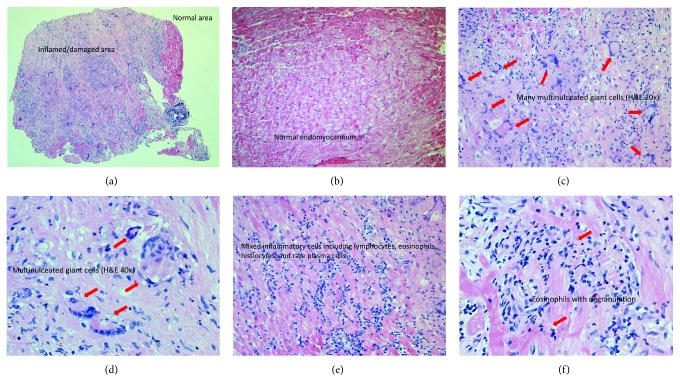
Pathological slides from the endomyocardial biopsy: (a) damaged and normal myocardium, (b) normal myocardium, (c) multinucleated giant cells (H&E staining 20x), (d) multinucleated giant cells (H&E staining 40x), (e) mixed inflammatory cells, and (f) eosinophils.

Right ventricular endomyocardial biopsy was performed. This demonstrated features typical of GCM, including extensive myocyte damage, multinucleated giant cells, and mixed inflammatory cell infiltrate. There was no granuloma formation (Figure 2). Autoimmune and connective tissue disease serology was unremarkable (negative anti-nuclear antibody, glomerular basement membrane antibody, anti-neutrophil cytoplasmic antibody, myeloperoxidase antibody, proteinase 3 antibody, and lymphotoxic antibody screening). Anti-heart autoantibodies were not tested on the patient and may be a limitation in the diagnosis of GCM.

The patient was treated with standard heart failure therapy including a beta-blocker, angiotensin receptor inhibitor, and mineral corticoid receptor antagonist. Once the diagnosis of GCM was confirmed, a combined immunosuppressive therapy with high-dose prednisone (60 mg daily), tacrolimus (alternating doses of 1 mg and 2 mg daily), and mycophenolate mofetil (1000 mg BID) was added. She also underwent implantation of a secondary prevention dual chamber ICD. In total, the diagnosis of GCM was established within 8 days of presentation, immunosuppressive therapy prescribed on day 9, and ICD implanted on day 14. Total hospital stay was 16 days, and there was no recurrence of VA or heart failure progression. She was discharged in stable condition and remained NYHA II at her follow-up appointments. Her echocardiogram at 6 and 12 months is unchanged with severe LV systolic dysfunction with minor regional variability and mild RV dysfunction. Repeat biopsy performed at 6 months demonstrated interstitial fibrosis and myocyte hypertrophy. Currently, our patient remains on her current immunosuppressive therapies aside from a tapering of her steroid dose to 5 mg daily.

## 3. Discussion

Idiopathic giant cell myocarditis (GCM) is presumed to be a T lymphocyte-mediated inflammatory disorder. An association with other autoimmune disorders such as thyroiditis and myasthenia gravis has been reported; however, it was found only among 20% of patients in a larger contemporary case series [2]. GCM affects men and women equally, with a mean age of onset of 42.6 years reported in the multicenter GCM study registry [2]. Common presentation includes heart failure and VA; however, it can also present as an acute myocardial infarction mimic or atrioventricular block [4].

Many cases are clinically diagnosed as idiopathic cardiomyopathy until autopsy or cardiac transplantation confirms GCM by histopathology [4]. Differential diagnosis includes sarcoidosis, but granulomata formation, which is absent in GCM, differentiates the two diseases. Repeated endomyocardial biopsy may be necessary to diagnose GCM, with higher sensitivity early in the disease process and in more fulminant cases with more extensive myocardial involvement [4, 5]. EMB has a reported sensitivity of 80% with a positive predictive value of 71% that increases with repeat biopsy [5]. Right ventricular septum was noted to be the target in most cases for myocardial sampling [4]. Imaging modalities such as echocardiography reveal reduced LV function and dilation. Contrast-enhanced cardiac MRI typically reveals areas of late gadolinium enhancement (scar) and increased T2-weighted signal (myocardial edema). ^18^FDG-PET shows the areas of perfusion defects and inflammation and may be utilized to target biopsies to the sites of active inflammation [4].

Prognosis is poor with a median survival of 3 months in the absence of treatment. Sole corticosteroid use was associated with an improved survival of 3.8 months. Combined immunosuppression may be more effective than corticosteroids alone with a median survival of 11.5 months with steroids plus azathioprine and 12.6 months with cyclosporine [2, 4]. GCM is known to recur in transplanted hearts with infiltrates identified on biopsy at a mean of 3 years posttransplant [2, 6]. There are limited data to guide long-term treatment strategies; however, it may be necessary to continue immunosuppression indefinitely due a relapse risk described even eight years after initial diagnosis [7]. Idiopathic GCM is an organ-specific autoimmune disease thought to be due to autoimmunity to myosin, thus necessitating an individualized immunosuppressive regimen with most cases requiring lifelong therapy.

Although seemingly effective, combined immunosuppression achieves partial clinical remission in only two-thirds of patients and EMB frequently shows ongoing inflammation. The lack of complete remission suggests the pathophysiology, and optimal treatment is not fully understood [3, 4, 8]. GCM recurrences have even been reported among patients who underwent heart transplantation [4, 9]. Despite medical therapy, sustained VA is present in up to 50% of patients and ICD implantation is recommended [2, 4].

Experimental models have suggested that there may be two forms of giant cell myocarditis with macrophage-derived giant cells and with myocyte-derived giant cells with the appearance of multinucleated giant cells corresponding to the fulminant phase of inflammation and myocardial damage [10]. Diagnosis is typically made via endomyocardial biopsy. Combined immunosuppressive therapy, implantable cardioverter defibrillators, and guideline-based heart failure therapies improve the overall prognosis; however, more research is needed to better understand this complex disease.

## References

[1] Kean B., Hoekenga M. T. (1952). Giant cell myocarditis. *The American Journal of Pathology*.

[2] Cooper L. T., Berry G. J., Shabetai R. (1997). Idiopathic giant-cell myocarditis — natural history and treatment. *New England Journal of Medicine*.

[3] Cooper L. T., Hare J. M., Tazelaar H. D. (2008). Usefulness of immunosuppression for giant cell myocarditis. *The American Journal of Cardiology*.

[4] Kandolin R., Lehtonen J., Salmenkivi K., Räisänen-Sokolowski A., Lommi J., Kupari M. (2013). Diagnosis, Treatment, and Outcome of Giant-Cell Myocarditis in the Era of Combined Immunosuppression. *Circulation: Heart Failure*.

[5] Shields R. C., Tazelaar H. D., Berry G. J., Cooper L. T. (2002). The role of right ventricular endomyocardial biopsy for idiopathic giant cell myocarditis. *Journal of Cardiac Failure*.

[6] Menghini V. V., Savcenko V., Olson L. J. (1999). Combined immunosuppression for the treatment of idiopathic giant cell myocarditis. *Mayo Clinic Proceedings*.

[7] Maleszewski J. J., Orellana V. M., Hodge D. O., Kuhl U., Schultheiss H.-P., Cooper L. T. (2015). Long-term risk of recurrence, morbidity and mortality in giant cell myocarditis. *The American Journal of Cardiology*.

[8] Cooper L. T., ElAmm C. (2012). Giant cell myocarditis. *Herz*.

[9] Gries W., Farkas D., Winters G., Costanzo-Nordin M. (1992). Giant cell myocarditis: first report of disease recurrence in the transplanted heart. *The Journal of Heart and Lung Transplantation*.

[10] Kodama M., Matsumoto Y., Fujiwara M. (1991). Characteristics of giant cells and factors related to the formation of giant cells in myocarditis. *Circulation Research*.

